# On Ribosome Load, Codon Bias and Protein Abundance

**DOI:** 10.1371/journal.pone.0048542

**Published:** 2012-11-07

**Authors:** Stefan Klumpp, Jiajia Dong, Terence Hwa

**Affiliations:** 1 Max Planck Institute of Colloids and Interfaces, Potsdam, Germany; 2 Department of Physics and Astronomy, Bucknell University, Lewisburg, Pennsylvania, United States of America; 3 Center for Theoretical Biological Physics, University of California San Diego, La Jolla, California, United States of America; Tel Aviv University, Israel

## Abstract

Different codons encoding the same amino acid are not used equally in protein-coding sequences. In bacteria, there is a bias towards codons with high translation rates. This bias is most pronounced in highly expressed proteins, but a recent study of synthetic GFP-coding sequences did not find a correlation between codon usage and GFP expression, suggesting that such correlation in natural sequences is not a simple property of translational mechanisms. Here, we investigate the effect of evolutionary forces on codon usage. The relation between codon bias and protein abundance is quantitatively analyzed based on the hypothesis that codon bias evolved to ensure the efficient usage of ribosomes, a precious commodity for fast growing cells. An explicit fitness landscape is formulated based on bacterial growth laws to relate protein abundance and ribosomal load. The model leads to a quantitative relation between codon bias and protein abundance, which accounts for a substantial part of the observed bias for *E. coli*. Moreover, by providing an evolutionary link, the ribosome load model resolves the apparent conflict between the observed relation of protein abundance and codon bias in natural sequences and the lack of such dependence in a synthetic *gfp* library. Finally, we show that the relation between codon usage and protein abundance can be used to predict protein abundance from genomic sequence data alone without adjustable parameters.

## Introduction

The genetic code maps sequences of nucleotide triplets or codons to sequences of amino acids. As the 20 amino acids are coded by 61 distinct codons, most amino acids are represented by multiple (2–6) synonymous codons. The nucleotide sequence of a gene therefore contains information beyond the amino acid sequence of the protein it encodes. This additional information is contained in the usage patterns of synonymous codons, which are not used in a random fashion, but with a bias towards a set of species-specific preferred codons [1], [2]. Extracting this additional information from sequence data is a key challenge for Systems Biology and numerous studies have linked the pattern of codon usage in gene sequences to various properties of the proteins such as their abundance in the cell [3], [4], [5], [6], their domain structure, folding kinetics, and cost of misfolding [7], [8], [9], and their evolutionary history, allowing, for example, to identify genes that have been acquired recently by horizontal transfer [10], [11].

On the mechanistic level, codon usage is known to affect the kinetics of translation as individual codons are translated at different rates [12], [13]. The differences in translation rates of synonymous codons are believed to result mostly from differences in the intracellular concentration of the corresponding tRNA species [13], [14], but small differences in the intrinsic kinetics have also been demonstrated [12], [15]. Moreover, differences in competition between binding of cognate, near-cognate and non-cognate tRNAs are also expected to contribute to the differences in translation speed [16]. Typically, codon usage is biased towards ‘fast codons’, as indicated by correlations between codon usage and abundance of the corresponding tRNA species [3], [13], [17] and by direct measurements of the (absolute or relative) translation rates of individual codons [12], [13].

In this study, we address the relation between the abundance of a protein and usage of synonymous codons in its genomic sequence. It has been observed long ago that the bias of codon usage is particularly pronounced in abundant proteins such as ribosomal proteins [4], [18], [19] and various indices measuring codon usage have been found to correlate with protein abundance in yeast and bacteria, indicating that codon usage may be used to predict protein abundance [5], [20], [21]. Also, optimization of codon usage often increases the yield of heterologous protein expression [22]. Therefore, the question arises whether there is a causal relation between the adapted, non-random codon usage and the expression level of a gene. In principle, such a relation should not be expected as long as translation of the gene is limited by the rate of initiation of translation. In that case, the synthesis rate of that protein does not depend on how fast a protein is translated but rather on how often ribosomes initiate translation of that protein. In agreement with this expectation, a recent study of a library of *gfp* variants that encode the same amino acid sequence with different nucleotide sequences, showed no correlation between the bias of codon usage and the resulting fluorescence intensity [23]. On the other hand, the same study showed that cell growth was impaired by expressing *gfp* sequences with many slow codons. This suggests that the biased codon usage provides a fitness advantage at a global level rather than at the level of the individual gene [23], as proposed earlier by Andersson and Kurland [14].

To elucidate the connection between codon usage in specific genes, protein abundance, and the growth state of the cell, we perform a comprehensive analysis of the hypothesis that biased codon usage is driven by the selection for low “ribosome load” in protein synthesis, i.e. for the efficient use of ribosomes through a preference for fast codons. Originally proposed by Andersson and Kurland [14], the hypothesis is based on the observation that ribosomes, the large machinery for protein synthesis, are the limiting commodity for rapidly growing cells. Not only do ribosomes participate in a large fraction of the biosynthetic activities during exponential growth, substantial experimental evidence, including the linear relation between ribosome concentration and growth rate [24], [25], [26], [27], direct observations indicating that protein synthesis is limited by the availability of free ribosomes [28], and observations that link the evolutionary costs of a protein to its synthesis [29], all indicate a propensity by fast growing cells to optimize ribosome usage. When the availability of free ribosomes (or their recycling after the termination of translation) limits protein synthesis, the use of fast codons should increase the ribosome recycling rate and thereby allow for a higher rate of overall protein synthesis and speed up cell growth. This picture, which is in line with earlier studies of codon usage evolution [30], [31], provides a natural explanation of the higher bias towards preferred (rapidly translated) codons in the gene sequences of abundant proteins: Every replacement of a fast codon by a slow one imposes a fitness cost by increasing the time for the ribosome to translate it. As this cost aggregates for every occurrence of the slow codon, i.e. every copy of the protein, the overall fitness cost of a slow codon is expected to increase with the abundance of the protein, in agreement with the observations that slow codons are very rare in the sequences of abundant proteins [3], [4], [18], [19].

However, if there is a fitness advantage favoring the use of fast codons, why are slow codons ever used, even in highly expressed ribosomal proteins? Moreover, the question arises whether codon bias and protein abundance can be related in a *quantitative* fashion? Answering these questions requires a quantitative assessment of the balance between this fitness advantage and the entropy cost of not using the slower synonymous codons. By performing such an analysis here through a mathematical model of evolution that incorporates selection of ribosome load through choices of codons and the associated fitness cost, we derive a relation between codon bias and protein abundance and test this relation using proteomic data for *E. coli*. This comparison indicates that the ribosome load hypothesis explains a substantial part of the observed codon bias and allows a quantitative comparison of the role of ribosome load for biased codon usage against other fitness advantages of codon bias. Most significantly, the quantitative relation between codon usage and protein abundance provides a simple means to estimate protein abundance solely from genomic sequences. Our estimate of protein abundance is seen to be positively correlated with the measured abundance data from proteomic studies with a correlation coefficient comparable to those between the abundance data from different experiments.

Two lines of research have previously addressed the relation between codon usage and protein abundance: On the one hand, statistical measures such as the ‘codon adaptation index’ have been developed and were shown to be correlated with protein abundance in bacteria and yeast, thus allowing to predict protein abundance semi-quantitatively from the patterns of codon usage in their genes [5], [20], [21]. These methods are typically based on observed correlations without reference to the mechanisms giving rise to those correlations, and the indices measuring codon usage do not necessarily have physical interpretations. On the other hand, aiming at a mechanistic understanding of codon usage bias, models for the evolution of codon bias have been developed that incorporate very detailed descriptions of the translation process and/or the evolutionary dynamics [31], [32]. Most recently, such detailed analysis, similar in scope to ours here, has been performed for yeast [32]. A relation between codon usage and protein abundance was used to determine codon-specific parameters of the underlying model such as translation rates for individual codons and biases in mutation rates from codon usage and protein abundance data. Such detailed models can generate excellent agreement with the data and allow one to extract detailed mechanistic parameters from given protein abundance and codon usage data. They are, however, less suited for predicting protein abundance, as they require a large number of parameters that need to be measured independently.

The approach we take here, a relatively simple evolution model and a simplified description of translation, attempts at combining the virtues of both types of previous approaches, sacrificing some of the accuracy gained by a more detailed mechanistic description. It is based on mechanistic pictures of evolution and translation, which, however, are simplified such that only sequence information is required to apply the model. In our view, the most significant consequence of establishing a quantitative relation between codon usage and protein abundance is that it provides a simple means to estimate protein abundance from genomic sequences. To that end, simple descriptions of codon evolution and of the dynamics of translation without unknown parameters are preferable, as they allow such estimates from genomic sequences alone, without the need for additional experimental information such as codon-specific translation rates, specific mutation rates, or tRNA concentrations.

## Results

### Relation between Codon Bias and Protein Abundance

To test the idea that ribosome load is the main driving factor for biased codon usage [14], we developed an evolution model (Eq. (6) in Methods) that describes the competition between mutation among synonymous codons and selection for the fast codons. The selection is defined [by Eq. (5)] as a dependence of the growth rate on the translation rates of individual codons (or the time a ribosome spends at each codon) [30]. The fitness cost of a slow codon is incurred every time this codon is read by a ribosome, and the overall fitness cost is proportional to the abundance of the protein encoded by the genetic sequence to which this codon belongs. The model thus describes a fitness landscape that decreases linearly from a single peak (sometimes called a Mount-Fuji landscape).

**Figure 1:**
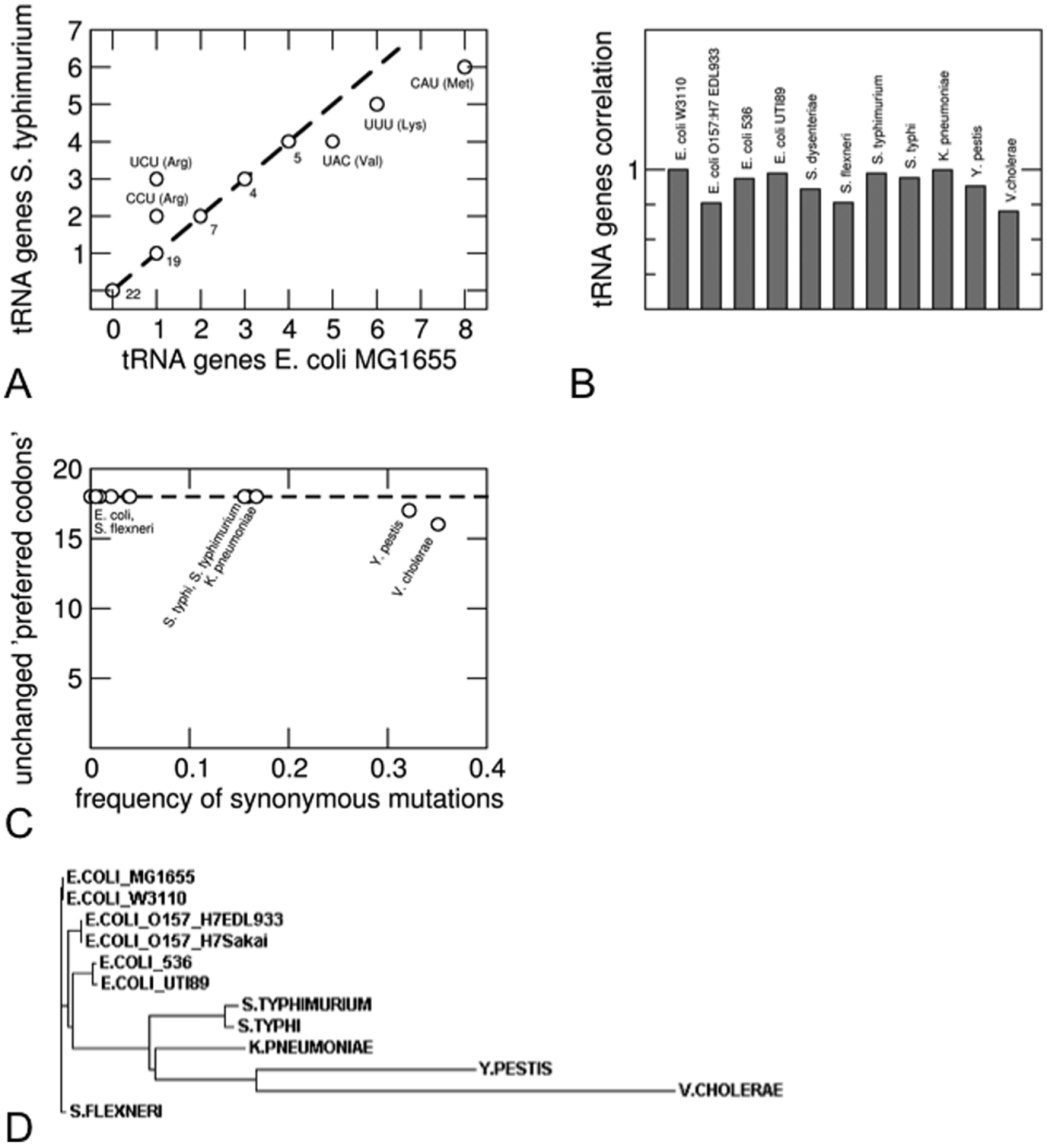
Evolution of tRNA concentrations and assignment of preferred codons. A: Correlation of tRNA gene copy numbers between *E. coli* MG1655 and *S. typhimurium*. For tRNAs with different gene copy numbers in the two organisms, the anticodon is indicated. B: Correlation coefficients of tRNA gene copy numbers of 11 enteric and related bacteria relative to *E. coli* MG1655. C: Number of unchanged ‘preferred codons’ as defined by Hershberg and Petrov [34] as a function of the frequency of synonymous mutations for the same 11 bacteria relative to *E. coli* MG1655. D: Phylogram obtained from an alignment of the *rpoB* sequences with ClustalW [60].

Before we discuss the relation between codon usage and protein abundance that results from our model, we note that, in principle, codon usage and the choice of the preferred codons (or the tRNA concentrations, which are a major factor determining that choice) co-evolve [30]. Our model does not account for such co-evolution, based on the following rationale: the shift from one preferred codon to another would affect many proteins and thus carries a much bigger fitness cost than individual synonymous mutations. We therefore expect codon preferences (and tRNA concentrations) to evolve on much longer timescales than the codon usage, so that the evolution of codon usage occurs essentially for fixed codon preferences. A similar argument has recently been supported by the observation that tRNA gene copy numbers (used as proxies for tRNA concentrations) are strongly correlated amongst different yeast species [33]. Here we analyzed the copy numbers of tRNA genes of 12 enteric and closely related bacteria. We found them to be highly correlated as well (Pearson correlation coefficients >0.7, Figure 1 A and B). Nevertheless, the tRNA gene copy numbers display a surprising amount of variability between closely related strains. We therefore considered a second measure of codon preferences, the ‘preferred codons’ for each amino acid as defined by Hershberg and Petrov [34], which are identified as those that get most strongly enriched with increasing codon bias among all genes of a species. For most of the bacteria considered here, the preferred codons are the same for all 18 amino acids despite a large fraction of synonymous mutations (Figure 1C). Differences in the preferred codons are only seen for the most distantly related species (Figure 1 C and D), which also exhibit the largest frequency of synonymous and non-synonymous mutations. These results support the general picture that the usage of synonymous codons evolves on a shorter timescale than the assignment of preferred codons.

As the translation rates are known only for a subset of codons, we classify all codons as either ‘fast’ or ‘slow’ according to their use in ribosomal protein genes (Table S1). This classification is clearly a simplification, but has the advantage that only genomic sequence information is required and no additional information such as cellular tRNA concentrations or measured translation rates for individual codons. This classification is consistent with the measured translation rates, where these are known, and it agrees with other classification schemes used in the literature (see Table S1 and Methods). Based on this classification, the fraction 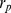 of slow codons in the nucleotide sequence of a gene arises as a natural measure of codon bias. 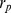 displays a simple dependence on the abundance of the protein (*N_p_*) encoded by that gene, that can be described by the interpolation formula.
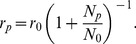
(1)


This dependence exhibits two regimes: For weak selection (low protein abundance), synonymous codons are used randomly, and slow codons occur with frequency *r*_0_, while for strong selection (protein abundance higher than a threshold abundance *N*_0_), the frequency of slow codons depends inversely on the protein abundance.

Even though it is known, *qualitatively*, for a long time that rare codons are particularly rare in abundant proteins [4], [19], we can now *quantitatively* compare the predicted dependence to genomic [35] and proteomic data [36], [37], [38], [39], [40] using *E. coli* as a model system. We analyze the relation between 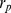 and 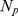 both at (i) the full protein sequence level using individual protein abundance and (ii) individual amino acid level (the next section) by examining the usage of synonymous codons for each amino acid across all measureable proteins in the organism.

**Figure 2:**
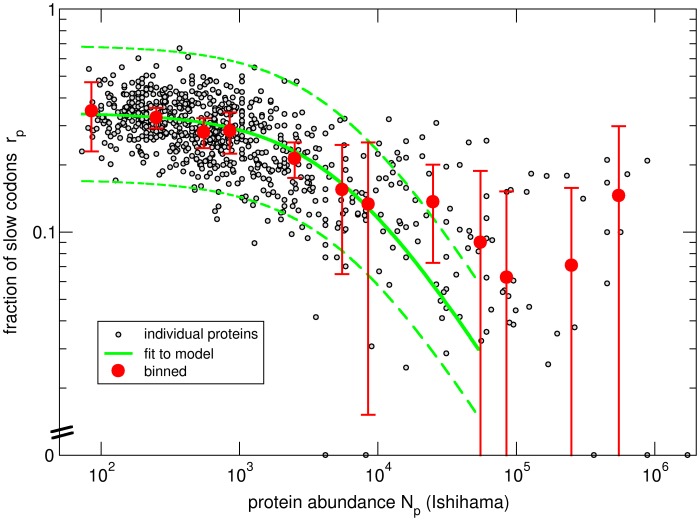
Relation between codon bias and protein abundance. Black dots show individual proteins with abundance data (N_p_, protein copy number per cell) taken from the data set of Ishihama et al. [36]. The red points depict averages over proteins of similar abundance (see the Supporting Text for a discussion of the error bars). The solid green line is fit of the model (Eq. 7) for individual proteins in the data set. The dashed green lines enclose a two-fold deviation from the fit. Codon bias is measured by the fraction of slow codons (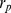) in the protein coding sequence accounting for all amino acids except Met, Trp, Glu, and Lys.

We compared the prediction from our model with five different protein expression data sets (Figures 2 and S1, Table 1). Figure 2 shows the comparison using the largest protein abundance data of Ishihama et al. [36]. Each dot in the plot represents one protein (through *N_p_* and *r_p_*), the red points show binned data, i.e. averages of *r_p_* over groups of proteins with similar abundance *N_p_* (within an up to 3-fold range of protein abundance), and the solid green line shows the prediction from the model for the best fit of the parameters 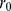 and 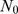. There is good agreement of the binned data with the model especially for low to medium protein abundance. At the same time individual proteins scatter widely (most within two-fold as marked by the dashed lines) around the predicted behavior. The deviation between the binned data and the model for very abundant proteins (*N_p_*>10000) could be specific to the abundance data of Ishihama et al., as it is not obvious in the other abundance data sets, which otherwise exhibit rather similar behavior (Figure S1). Abundance values for high-abundance proteins are generally somewhat uncertain, as indicted by the wide range of abundance numbers for different ribosomal proteins (which should have the same abundance in the cell), but this scatter is particularly pronounced in the Ishihama et al. data set. The protein abundance at which strong selection sets in, characterized by the parameter 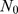 in Eq. (1) is found to be a few thousand molecules per cell (*N*_0_≈5030 with the data of Ishihama et al.; the precise value depends on which protein abundance data set we use, see Table 1). This value of *N*_0_ falls within the range obtained from microscopic parameter estimates (see Methods). For low protein abundance, our fit leads to 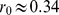. This is lower than the expected value based on fully random usage of synonymous codons (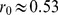, see Methods), providing a first indication that not all codon bias is captured by the ribosome load hypothesis.

**Table 1:** Data sets for protein abundance and parameters of fit with the abundance-codon bias relation from Eq. (6).

Data set	Growth conditions	Method for quantification	Number of genes/proteinsincluded in our analysis*	Parameter values fromfit with Eq. (1)	
				*r_0_*	*N_0_*
Ishihama et al. [36]	Data for rich medium andminimal medium (glucose +amino acids) pooled	emPAI (LC-MS/MS)	798	0.34	5034
Lu et al. [37]	MOPS glucose	APEX (LC-MS/MS)	426	0.30	12181
Link et al. [39]	MOPS glucose	2d gel, staining intensity	120	0.26	2964
Lopez-Campistrous et al. [40]	MOPS glucose	2d gel, staining intensity	380	0.33	7701
Pedersen et al. [38], withadditional proteinidentification [63]	Rich defined medium	2d gel, 2 radioactive labels	69	0.29	4097

* Proteins for which annotations did not match between data sets were excluded from our analysis, as were proteins of length <50 amino acids.

For comparison, we also used a gene expression data set from an RNA microarray experiment (from ref. [41], data for rich medium) and analyzed it in the same way (Figure S2). Using mRNA levels as a proxy for protein abundance has the disadvantage that it implicitly assumes that all genes are translated at the same rate, while in reality there is a broad distribution of the ratio of proteins per mRNA [37]. Indeed the main difference to the results with proteomic data (Figs. 2 and S1) is the even greater scatter of the fraction of slow codons for highly expressed genes.

### Analysis for Individual Amino Acids

**Figure 3:**
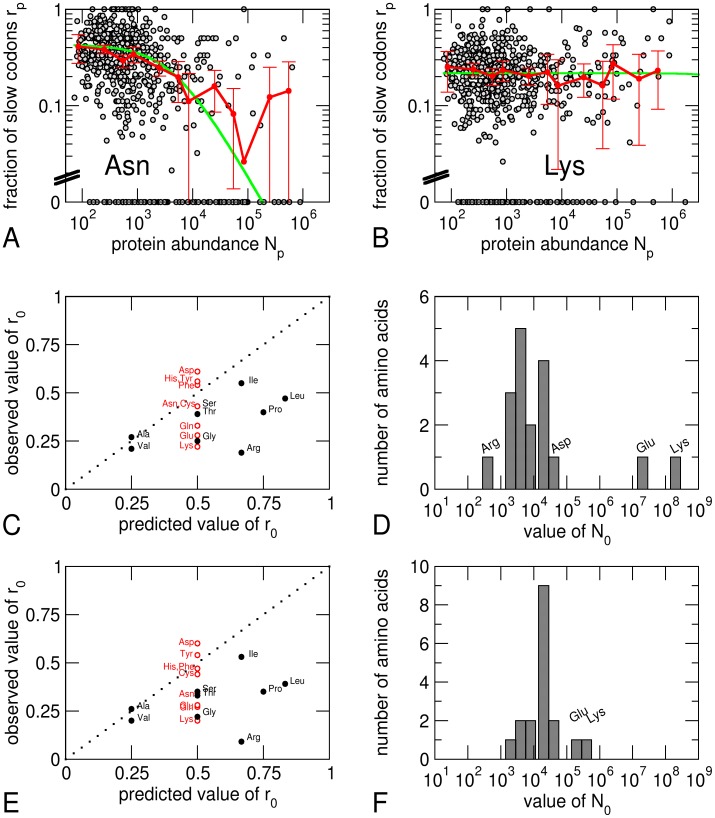
Analysis of individual amino acids for codon usage bias and protein abundance. A and B: Fraction of slow codons (*r*_p_) for Asn and Lys plotted against the protein abundance [36]. As in Figure 2, black dots show data for individual proteins; red dots show averages over proteins of similar abundance; the solid green line is a fit based on Eq. 7. Dashed green lines mark the two-fold range from the model to the measured protein abundance. C: Values of *r*_0_, the parameter characterizing codon usage bias in the weak-selection limit plotted versus the expected values based on the assumption of complete random codon usage. Red dots are for amino acids encoded by two synonymous codons, black dots for those with more than two synonymous codons. D: Histograms of values obtained for the threshold parameter *N*_0_ that characterizes the protein abundance above which strong selection sets in. Abundance data in (C) and (D) are from Ishihama et al. [36]. (E) and (F) contain the same measures as (C) and (D) using abundance data from Lu et al. [37].

We also performed the same analysis for each individual amino acid encoded by more than one codon (i.e. excluding Trp and Met). Figs. 3A and B show two examples, Asn and Lys (discussed below); results for all amino acids are shown in Figure S3. For every amino acid, we determined 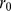 and 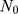 (Fig. 3C–F) by fitting with Eq. (1) using the two abundance data sets of Ishihama et al. [36] and Lu et al. [37]. We compared the values of 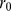 (which measures the bias of codon usage for low abundance proteins, i.e. in the weak selection limit of our model) as obtained from the fit with the expectations from random usage of synonymous codons. The expected and observed values are strongly correlated, but one can see that the 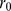 values from the fit tend to be smaller than expected (Figure 3 C and E). This indicates that codon usage is not fully random even for low-abundance proteins.

The parameter 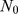 indicates the threshold in protein abundance where selection becomes dominant. For most amino acids, 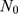 is found to be in the range of a few 1000 proteins/cell (Figure 3 D and F). The fact the values for 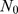 are similar for all amino acids provides a rationale for pooling different amino acids together. An exception are the amino acids glutamate and lysine, for which we find essentially no or only very weak abundance-dependence of codon bias (Figures S3A and 3B), corresponding to much larger *N*_0_ than for other amino acids (Figure 3 D and F). Both however exhibit abundance-independent codon bias, with 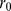 values of 0.28 (Glu) and 0.22 (Lys). For this reason, Glu and Lys were excluded in the calculations of the fraction of slow codons (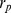) of Figure 2 and Figure S1.

### Effects of Sequence Length

**Figure 4:**
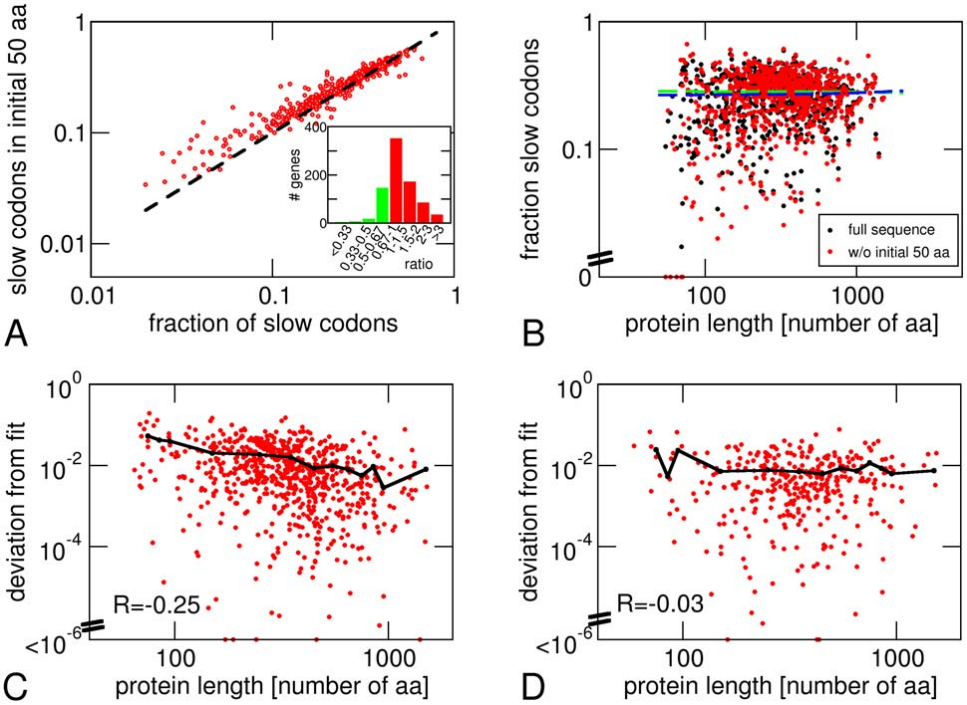
Analysis of sequence-length dependent effects. A: Fraction of slow codons (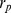) in the initial part of a gene sequence (first 50 amino acid) vs. fraction of slow codons in the rest of the sequence. Inset: Histogram of the ratio of these two fractions of slow codons. B: Dependence of the fraction of slow codons on the sequence length with (black) and without (red) the initial 50 amino acids. C and D: Length-dependence of the mean square deviation of the codon usage in a protein sequence from expected values based on the fitted average, using the abundance data of Ishihama et al. [36] (C) and Lu et al. [37] (D).

In the analysis shown so far, we have excluded the first 50 amino acids of each protein sequence because several studies have found that the initial part of coding sequences is enriched in rare codons [42], [43], a phenomenon attributed to either a requirement for weak secondary structures in the mRNA [23] or the need for a slow on-ramp to limit the maximal translation rate and prevent ribosome traffic jams [43], [44]. Our analysis confirms this observation: We find that for most genes, the fraction of slow codons (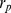) is slightly (<2-fold) higher in the initial sequence than in the rest of the sequence (Figure 4A). Overall, we do not see a dependence of the fraction of slow codons (*r*_p_) on the length of the protein sequence, both for full sequences and for sequences without the first 50 amino acids (correlation coefficients *R* = −0.01 and 0.02, respectively, see Figure 4B).

The observation that binning the proteins with similar abundance reduces the scatter of the data and improves the agreement of the data with the model suggests that such scatter in individual proteins may simply be due to insufficient sampling as the sequences of individual proteins may be too short. Accordingly, the deviation of the observed fraction of slow codons (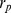) from the prediction should be lower for larger proteins (longer amino acid sequences). We proceeded to calculate the mean square deviation between the data and model prediction for each sequence and tested for correlations with the sequence length (Figure 4C and D). However, we found only very weak negative correlation (correlation coefficients *R* = −0.25 and −0.03 for the data sets of Ishihama et al. [36] and Lu et al. [37], respectively). We therefore conclude that the deviations of the fraction of slow codons (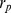) observed in individual proteins from the predictions of our model are not random variations due to limited sampling, but rather reflect some other properties of these proteins or their mRNA not captured by our model (see also the calculation of error estimates in Methods). Nevertheless, these deviations are averaged out if proteins of similar abundance are pooled together.

### Predicting Protein Abundance

**Figure 5:**
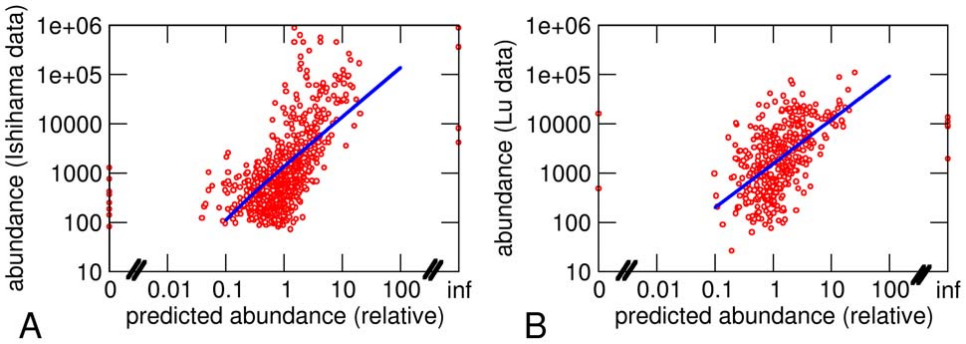
Predicting protein abundance from sequence data. Predicted protein abundance (relative to the selection threshold *N*_0_) as obtained from Eq. 2, vs. the measured abundance data of Ishihama et al. [36] (A) and Lu et al. [37] (B).

Finally, the relation between the frequency of slow codons and protein abundance that results from the evolution model can be used to predict protein abundance from its gene sequence. Our analysis suggests the following procedure: First, codons have to be classified as either fast or slow according to their usage in ribosomal protein genes as described in Methods. No information beyond sequence information (such as tRNA concentrations) is needed. Second, the fraction of slow codons, 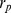, in each protein is determined. The predicted abundance of the protein can then be obtained from rewriting Eq. (1) as.
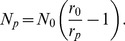
(2)provided that estimates for 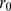 and 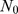 are known. However 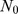, for which only a relatively broad range can be estimated (see Methods) is only required for absolute protein copy numbers, as 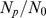 gives protein abundance relative to the abundance threshold for strong selection. *r*_0_ can be estimated from random codon usage, weighted with the amino acid frequencies (for *E*. *coli*, this leads to 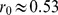, see Methods). We predicted the abundance for all *E. coli* proteins and compare them with the abundance data from Ishihama et al. [36] and Lu et al. [37] in Figure 5A and B. The two quantities are clearly correlated with correlation coefficient *R* = 0.66 and *R* = 0.54, respectively (calculated excluding all proteins for which the predicted abundance values are negative or infinite). The correlation is comparable to the correlations among different published abundance data sets (e.g., Ishihama et al. – Lu et al.: *R* = 0.62, Ishihama et al. – Pedersen et al.: *R* = 0.69, see Table S2).

## Discussion

In this study we have analyzed biased codon usage in fast growing bacteria based on a global fitness criterion: the efficient use of the translation machinery and the rapid recycling of ribosomes, which are a growth-limiting commodity in fast-growing bacteria (and, possibly, also in rapidly proliferating eukaryotic cell) [14]. According to our model, usage of fast codons is advantageous for the overall growth of the cell. It reduces the time during which ribosomes are sequestered in translation and thereby increases the concentration of free ribosomes and speeds up initiation of translation. This hypothesis provides a natural explanation of the observation that codon usage is more strongly biased in abundant proteins, as the fitness cost associated with each slow codons is higher for abundant proteins, while also giving a rationale and quantitative guideline on how codon bias is diminished in less abundant proteins.

By formulating this idea in mathematical terms, we were able to obtain a general relation between protein abundance and the frequency of slow codons (Eq. 6). We want to emphasize that such relation as obtained from the ribosome load hypothesis is not a causal relation, but a result of evolution. Therefore it is not surprising that a library of *gfp* variants with the same amino acids sequence, but different nucleotide sequences [23] does not exhibit such a relation (but with consequence on poor cell growth).

Contrary to this picture, optimizing codon usage often increases the yield of heterologous gene expression [22], [45], an observation that suggests a causal relation between between codon usage and protein abundance. In many cases, this link may again be a global one, e.g. if overexpression of a sequence rich in rare codons depletes the cellular pool of one or several rare tRNAs [22]. Such global effects do not contradict the ribosome load picture, but rather add another layer of selection pressure that comes into play for very large protein abundance or strong overexpression of sequences with many rare codons. Likewise, the translation sequences with a large number of slow codons may become limitated by elongation rather than initiation. In such cases, which are not expected to be typical for native (evolved) sequences [14], but may be common in heterologous expression (a systematic study of luciferase genes with different codon usage in yeast [46] possibly provides an example), gene expression is influenced by codon usage in a local, gene-specific fashion. As a caveat, we note that if such local mechanisms are dominant in an organism, ribosome load may not be the major driving force of codon usage bias.

We have tested the relation between codon bias and protein abundance obtained from the ribosomal load hypothesis using proteomic data for *E. coli* and found good agreement for binned data, in which proteins of similar cellular abundance are pooled together. Individual proteins, however, show considerable scatter around this average behavior (Figure 2 and Figure S1). Furthermore, our analysis indicates that these deviations are not simply the result of limited sampling in short sequences (Figure 4 C and D). This observation indicates that codon usage has other roles in addition to reducing ribosome load. The same conclusion can be drawn from the observation that codon usage is not completely random even for low abundance proteins (Figure 3 C and E). Indeed a number of such roles have been proposed. For example, codon usage may also be used to optimize the accuracy of translation as indicated by a more biased codon usage in highly conserved amino acids [47], [48]. Moreover, some slow codons may be important for the proper co-translational folding of a protein or for the formation of mRNA secondary structures that are accessible for the ribosome [8], [23]. Such effects are likely to impose a selection pressure towards keeping specific slow codons in place that does not depend on protein abundance (such codons are likely to occur at a fixed frequency in protein-coding sequences, as we found no dependence of codon usage on protein length, see Figure 4). Such specific selection may counterbalance the general fitness costs of slow codons. Nevertheless when we average over proteins of similar abundance, we obtain good agreement with the predicted trend. This observation can be interpreted in the following way: If a slow codon is required at a specific sequence position for a specific reason such a RNA structure, the general fitness cost due to ribosome load created by that slow codon can be offset by replacing a slow codon by a fast codon in a different sequence position, where no selection for slow codons exists. This position may either be in the same gene or in a different gene encoding a protein of the same abundance. In fact, within our model, fitness is determined by the sum of the fitness costs of slow codons in all protein-coding sequences; there is no need for adapting codon usage in every sequence individually. It is possible that part of the deviations from the predicted trend that we see for the most abundant proteins (at least in some data sets with the most pronounced effect in the Ishihama et al data) is a consequence of such specific selection for slow codons, as such offsetting may not always be possible in these sequences, where slow codons are already rare.

Finally, our analysis also points out a simple strategy for the prediction of protein abundance from sequence data alone. This requires two major steps, a classification of codons as either fast or slow by any suitable criterion, and the calculation of the frequency of slow codons in a protein-coding sequence, from which the abundance of that protein is obtained via a rather straightforward Eq. (6). Here we used the frequency of a codon in ribosomal protein genes as the classification criterion. This criterion is advantageous as only genomic sequences are required and no additional information (e.g. tRNA concentrations) is needed, but other criteria are clearly possible. Compared to earlier methods for the prediction of protein abundance from statistical analysis of codon usage [5], [20], our approach has the advantage of being based on a microscopic physical picture, which is however simple enough that it does not require a large number of parameters that need to be determined independently. It should therefore be possible to improve its predictions by including additional, abundance-independent sources of codon bias into the underlying model. One way of improving the agreement of our model with the data and thus the accuracy of predicted protein abundance would be to identify slow codon that are kept in place by positive selection, e.g. because they have a role in protein or mRNA folding. Such codons will clearly contaminate the analysis done we and if they can be identified (e.g. via their conservation across species), excluding them from the analysis should reduce the scatter of individual proteins around the average behavior and improve the predicetion of protein abundance.

Finally, we want to point out that the arguments used in this study also apply to other sequence-dependent mechanisms of slowing translation. Specifically, a recent ribosome profiling study has questioned the importance of specific codon usage for the overall speed of translation (that had been demonstrated by earlier direct measurements [12], [13]), but rather points towards internal Shine-Dalgarno-like sequences as the major source of pausing during translation [49]. The ribosomal load hypothesis applies equally to slowing of translation by Shine-Dalgarno-like pause sequences. As a consequence, we expect stronger selection against such sequences in highly expressed proteins, and thus it should be possible to derive a quantitative relation between the frequency of such sequences and protein abundance, similar to the one for slow codons derived here.

In summary, we have analyzed codon usage based on the hypothesis that ribosome load is a major determining factor of codon bias. This idea is based on observations that ribosomes are a growth-limiting commodity under rapid cell growth. Our analysis of codon usage in *E. coli* shows that this idea can explain a substantial part, but not all, of the observed codon bias.

## Methods

### Fitness Landscape

In a fast growing unicellular organism, a characteristic fitness measure is the growth rate *μ*. The effect of translation speed on *μ,* can be calculated by the following argument given first by Bulmer [30], [31]: If ribosome load is growth limiting in the sense that essentially all ribosomes are translating all the time, then the doubling time τ of the cells is given by the time these ribosomes need to double all protein components of the cell,
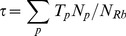
(3)


In this equation, the sum runs over all proteins in the cell. *T_p_* is the time it takes to translate one single protein *p*, *N_p_* is the copy number of this protein, and *N_Rb_* is the number of ribosomes per cell. (If there is extensive turnover of a protein, the number of copies of that protein that are synthesized during one cell doubling can be considerably larger than the abundance of the protein in the cell. In that case, 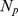 should be interpreted as the number of copies synthesized during a cell doubling or as the synthesis rate. However, under conditions of rapid cell growth that are relevant here, proteins are typically stable in *E. coli* [50], [51].) We assume that every change from a fast codon to a slow codon increases *T_p_* by a small time Δ*t,* which in principle can be codon-dependent (as, e.g., in Ref. [32]). As will be justified below, we will make the simplification that all codons can be classified as either fast or slow and that there is only one such translation time difference.

We now consider a single protein and write the doubling time τ as a function of the number *n* of slow codons in this specific protein as
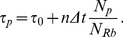
(4)


The growth rate is
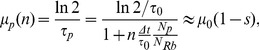
(5)where τ_0_ and μ_0_ = ln2/τ_0_ are the doubling time and growth rate in the absence of slow codons for the protein under consideration and 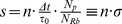 is defined as *the selection coefficient*. The approximation in Eq. (5) requires 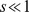 (shown to be the case below) and leads to a linear, Mount Fuji-type fitness landscape with a selection coefficient, *s*, that is proportional to the abundance of the protein under consideration.

### Evolution Model

We now use the expression for fitness given in Eq. (5) in an evolution equation [52] for a population of cells with different codon usage for a particular protein sequence. Assuming all codons are either fast or slow, we write the evolution equation for the number of cells in that population that have *n* slow codons in the sequence of a particular protein *p*, *N*(*n*), as
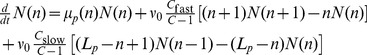
(6)


The first term on the right hand side describes growth, the second and third term describe beneficial and harmful mutations by decreasing and increasing *n*, the number of slow codons, respectively. ν_0_ is the synonymous mutation rate per codon. *C* is the total number of codons encoding the amino acid under consideration, so *C*-1 is the number of possible mutations. *C_fast_* and *C*_slow_ = *C*- *C*_fast_ are the number of fast and slow codons. *L_p_* is the total number of codons (length of the protein *p*).

Several comments are in order: (i) In Eq. (6), *N*(*n*) is not normalized to a fixed population size. The equation describes an overall exponentially growing population and is therefore linear. It can thus be solved as an eigenvalue problem that has one positive eigenvalue corresponding to exponential growth of the total population while the distribution of the number of fast and slow codons in the population is stationary. (ii) Here we consider a deterministic evolution equation, thus neglecting genetic drift. For the case *C*_fast_ = *C*_slow_ = 1, the stochastic effects resulting from a finite population size *N*_pop_ have been studied previously. The finite population size was found to effectively modify the mutation rate ν_0_ to ν_0_+1/(4*N*_pop_) [53]. (iii) The main parameter of the model is 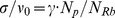, where 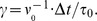 We provide an estimate of 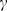 for *E. coli* below.

### Solution of the Evolution Equation

Within our model of codon usage evolution, the fraction 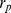 of slow codons (or the probability that a given codon is a slow one) in a protein emerges as the natural measure for the usage of synonymous codons. This quantity is calculated from the average number of slow codons 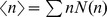 via 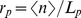. As codons are independent in our model, 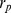 can be calculated by considering a single codon, which leads to
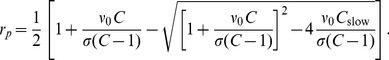
(7)


We note that for *C*_fast_ = *C*_slow_ = 1, the exact solution has been given previously [30], [53]. In the limit of weak selection (small 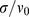 or low protein abundance), 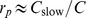, which corresponds to random usage of synonymous codons. For strong selection, 
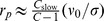
. Throughout our analysis we use the simpler expression given by Eq. (1) that interpolates between the two limits (plotted in Fig. S4 together with the exact result). It depends on two parameters, 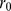 and 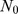, that describe the codon usage under weak selection and the threshold for protein abundance-dependent selection, respectively. They can be related to the microscopic parameters of our model via the two limiting cases of weak and strong selection, from which we obtain 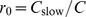 and 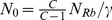 (where we have used the relation 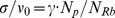).

These relations are used to estimate expected values for both *N*_0_ and *r*_0_: In principle, there is one value of *r*_0_ for every amino acid, as *r*_0_ depends on *C*_slow_ and *C*, the numbers of slow and all codons encoding that amino acid (Fig. 3C and E and Table S1). However, we can get an overall estimate for *r*_0_ by averaging these values, weighted with the frequency of the amino acids in all protein-coding sequences in the *E. coli* genome. This leads to *r*_0_≈0.53. Likewise we can estimate an overall threshold value for selection by ignoring the prefactor *C/(C-1)*, This leads to values of *N*_0_ that are about 1/10 of the abundance of ribosomes (using 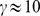). With a ribosome abundance of several 10 0000 molecules per cell [26], this corresponds to an *N*_0_ value of several thousand molecules per cell. This estimate is, however, strongly dependent on the estimate of γ for which we can only estimate a rather wide range (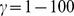, see below).

### Estimate of the Model Parameters

The main parameter of our evolution model is 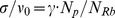, the ratio of the selection coefficient and the mutation rate, which is proportional to the abundance of the protein under consideration. The dependence of this parameter on the protein abundance is characterized by 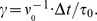 ν_0_ can be estimated by the mutation rate per base which is about 10^−9^ per generation [54], under the approximation that all third-position mutations are synonymous. A naïve estimate of the selection parameter, known to overestimate the selection strength [30], is obtained using the doubling time τ_0_∼1 hour and taking the translation time difference between synonymous codons to be 10% of the average translation time per codon (∼0.1 seconds [12]). This leads to γ ∼ 10^3^. This value strongly overestimates the strength of selection mainly because in natural environments, fast exponential growth is not typical. However, selection for fast translation is likely present only during rapid cell doubling. We can therefore improve the estimate by multiplying it with the fraction of total time that the cells actually grow fast. A rough estimate is a few hours per a few days or more [55], [56], so γ would be reduced by a factor of 0.001–0.1. We thus expect to find values of γ in the range 1–100. These estimates of γ also imply that our assumption of 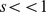 is fulfilled.

Alternatively γ can be estimated from codon usage data using the results of our model: One option is to use the sequences of ribosomal proteins (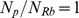) and to determine γ from their average fraction of slow codons. This procedure requires no proteome data and leads to γ≈10. A second option is to obtain γ from our fit of the model to the dependence of codon usage on protein abundance (see Results) via 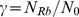. From that analysis we obtained γ≈1.4 using the abundance data of Lu et al. [37] and γ≈100 using the abundance data of Ishihama et al. [36], both in the range obtained from the microscopic estimate given above. The difference between the two values is mostly due to the different abundance of ribosomal proteins in the two data sets.

### Co-evolution of Codon Usage and Codon Preferences (or tRNA Concentration)

To test the hypothesis that evolution of codon preferences or tRNA concentration occurs on larger time scales than the evolution of the usage of individual codons, we obtained tRNA gene copy numbers from the database tRNADB-CE [57] for 12 enteric bacteria and determined the correlations between. Even though tRNA gene copy numbers are not a direct measure of tRNA concentrations or codon preferences, they are correlated [17], [58]. Nevertheless, there are some difficulties associated with the use of tRNA gene copy numbers. In particular, we do not find a systematic variation of the correlation coefficients plotted in Figure 1B with the phylogenetic distance or the frequency of mutations. The latter was estimated by aligning *rpoB* sequences [a commonly used protein-coding alternative to ribosomal RNA sequences for phylogeny and ecological analysis [59]] using ClustalW [60], [61] and counting synonymous and non-synonymous mutations. Therefore, we also counted changes in the ‘preferred codons’ for each amino acid as defined by Hershberg and Petrov [34], a measure of codon preference that requires only genomic sequence information.

### Identification of the Fast and Slow Codons

As mentioned above, the time difference Δ*t* could in principle be measured for each synonymous substitution. However, either absolute or relative speeds of translation have only been measured for a small set of codons [12], [13]. For that reason, we adopt a simplified description in which all codons are classified as either fast or slow according to their usage in the gene of ribosomal proteins, which are known to exhibit strong codon bias [4], [18], [19]. Codons that are preferentially used in ribosomal protein genes are considered as fast codons (Table S1). Compared to other criteria [34], [44], [62] such as classifications according to tRNA concentrations, this criterion has the advantage that it depends only on sequence information and does not require the knowledge of tRNA concentrations or translation speeds. It is therefore readily applicable to a species once its genome is sequenced.

Alternative classifications are based on tRNA concentrations (or tRNA gene copy numbers). There are, however, several difficulties in using tRNA concentrations as a measure of the translation speed of a codon: First, two codons recognized by the same tRNA may be recognized with different affinities. This possibility is indicated by the observation that for 7 out of the 9 amino acids encoded by 2 codons (Asn, Asp, Cys, Glu, His, Lys, Phe), there is only one tRNA that reads both codons, yet there is still a clear “preferred” one between the two. In all of these cases, the preferred codon is the one with perfect codon-anticodon matching (Table S1). Second, it has been shown *in vitro* that kinetic parameters of the ribosome are codon-dependent even when corrected for tRNA concentrations, although tRNA concentration is likely to account for the biggest effect [15]. Furthermore, if a codon is read by several tRNAs, their concentrations would have to be weighted with the corresponding affinities. Nevertheless, tRNA concentrations are known to correlate with codon preferences [3], [17] and the choice of fast codons according to our criterion of use in ribosomal genes mostly agrees with the choices obtained based on other criteria (Table S1). In particular, we emphasize that whenever we identify a single preferred codon, it agrees with the ‘optimal codon’ in Ikemura’s codon hierarchy [62], which is based on tRNA concentrations together with structural arguments, and with the bioinformatics-based ‘preferred codon’ of Hershberg and Petrov [34]. When we classify several codons encoding the same amino acids as ‘fast’, the ‘preferred’ or ‘optimal’ codon according to these two approaches is always found among our ‘fast codons’, although it is not always the one that is used most frequently in ribosomal protein genes (Table S1).

### Average Fraction of Slow Codons and Error Estimates

The relation between protein abundance (*N_p_*) and the fraction of slow codons (*r_p_*) provided by our evolution model applies also to individual codons if one interprets *r_p_* as the probability that a certain codon is a slow codon. Therefore the average fraction of slow codons (e.g., the red points in Figure 2) in a group of proteins of similar abundance can be determined in two ways: We can take the point of view of the proteins and calculate the (weighted) average *r_p_* value over these proteins, 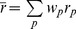
with the weights 
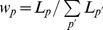
given by the sequence length *L_p_*. Alternatively, we can calculate the fraction of slow codons in the set of all codons of these sequences as an estimate of the probability that such a codon is a slow codon. In that case, we treat the sequences of these proteins as if they were one long sequence and discard all information about the identity of proteins. Because we weight sequences by their length, the two calculations lead to the same result for the average fraction of slow codons. However, they result in different estimates of the accuracy of that average (Table S3). In the first case, we calculate a (weighted) mean of a set of *r_p_* values that exhibit a certain distribution. The standard error of that mean (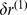) reflects the standard deviation of that distribution and is given by
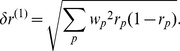
(8)


This error estimate is indicated by the red error bars in Figures 2, 3 and S1. From the second point of view, we determine a fraction of codons that are slow among a set of codons. The standard error of that fraction is obtained as
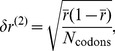
(9)where 
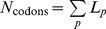
 is the total number of codons from all sequences. This error estimate is smaller than the first one (Table S2) and is comparable to the size of the red circles in Figure 2. We interpret the difference between these two error estimates as reflecting the fact that the deviations of the fractions of slow codons in the sequences of individual proteins from the average is not due to random sampling, but due to other roles of codon usage, as also suggested by the lack of a length dependence (Figure 4C and D).

## Supporting Information

Figure S1 **Relation between codon bias and protein abundance for several abundance data sets.** The same quantities are plotted as Figure 1 using protein abundance data from Lu et al. [37], Link et al [39], Lopez-Campistrous et al. [40] (A), and Pedersen et al. [38] (B). In (B), the dashed green lines indicate two-fold deviations from the fit of the model to the individual protein data. Genes with more than two-fold deviations are labeled.
(TIFF)

Figure S2 **Relation between codon bias and mRNA levels.** mRNA levels (data for growth in LB medium from ref. [41]) are used as a proxy for protein abundance. The data is analyzed in the same way as in Figure 1.
(TIFF)

Figure S3 **Relation between codon bias and protein abundance analyzed for individual amino acids.** The same quantities are plotted as in Fig. 2, using again abundance data from Ishihama et al. [36]; however for each panel only codons encoding one particular amino acid were taken into account. A: amino acids encoded by two codons, B: amino acids encoded by more than two codons.
(TIFF)

Figure S4 **Exact solution of the evolution model and interpolating approximation.** The calculated fraction of slow codons (*r*_p_) is plotted as a function of the selection coefficient σ (which is proportional to the protein abundance and scaled here with respect to the mutation rate ν_0_) as obtained from the full solution (Eq. 7, solid lines) and the approximation given in Eq. 1 that interpolated between the limits of weak and strong selection (dashed lines). The two cases are for an amino acids encoded by 1 fast and 1 slow codon (black) and 3 fast and 1 slow codon (blue).
(TIFF)

Table S1 **Preferred codons.**
(PDF)

Table S2 **Correlations between measured and predicted protein abundance and between different abundance data sets.**
(PDF)

Table S3 **Error estimates for the average fraction of slow codons.**
(PDF)
