# Supplementary figures and images for: On Ribosome Load, Codon Bias and Protein Abundance
Source: PLoS One. 2012 Nov 7;7(11):e48542. doi: 10.1371/journal.pone.0048542 (PMC3492488; doi:10.1371/journal.pone.0048542)

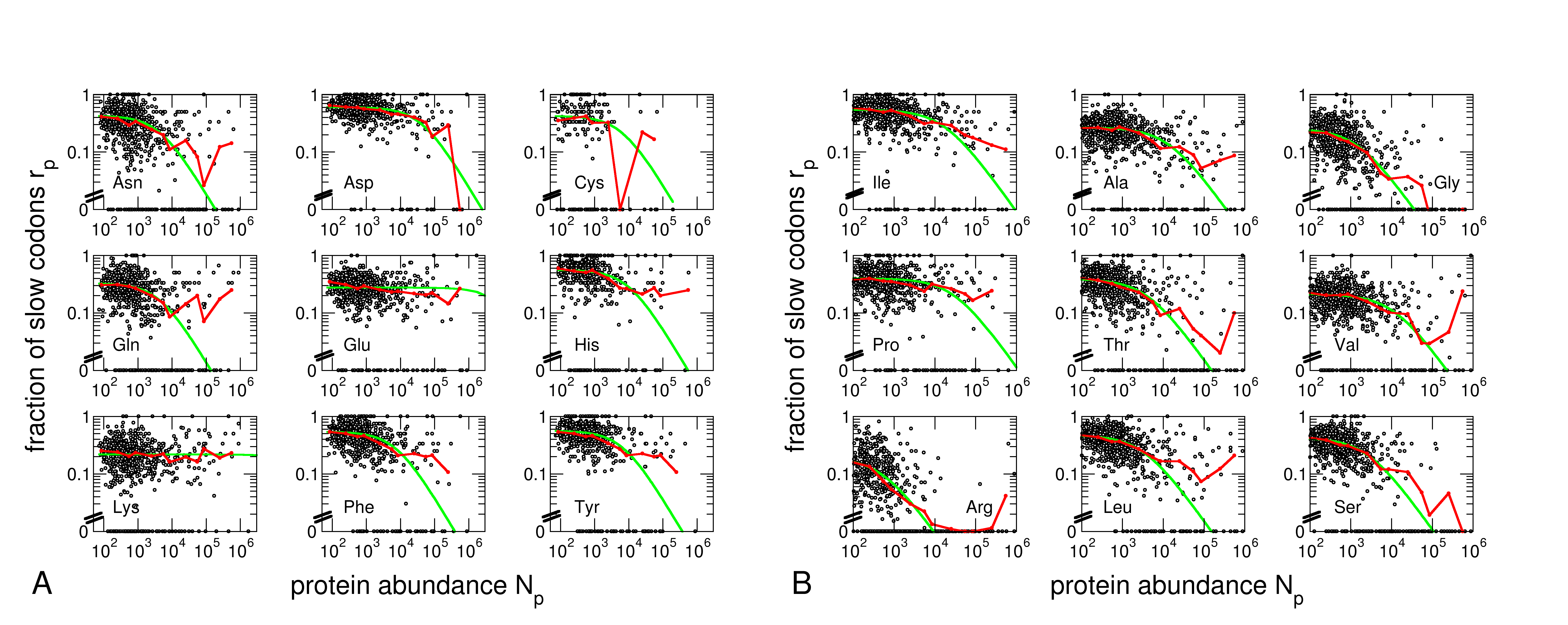

Supplement: Figure S3 — Relation between codon bias and protein abundance analyzed for individual amino acids. The same quantities are plotted as in Fig. 2, using again abundance data from Ishihama et al. [36]; however for each panel only codons encoding one particular amino acid were taken into account. A: amino acids encoded by two codons, B: amino acids encoded by more than two codons. (TIFF) [file pone.0048542.s003.tif]
