# Supplementary material for: On Ribosome Load, Codon Bias and Protein Abundance
Source: PLoS One. 2012 Nov 7;7(11):e48542. doi: 10.1371/journal.pone.0048542 (PMC3492488; doi:10.1371/journal.pone.0048542)
Supplement: Table S1 — Preferred codons. (PDF) [file pone.0048542.s005.pdf]

Table S1: Preferred codons

Amino acid	Codons	Translation speed		tRNA gene copy number (b)	tRNA concen-tration [μM] (c)	Codon usage in the proteome (d)	Codon usage in r-protein genes	Codon hierarchy (Ikemura, 1981) (e)	Preferred codons (Hershberg and Petrov, 2009)	Taken as preferred in our analysis
		Sorensen and Pedersen (1991) [sec^{-1}]	Curran and Yarus (1989) (relative) (a)							
Asn	AAC<br>AAU			4<br>0+4	7.29<br>7.29	29.21<br>5.61	30.7<br>4.8	AAC> AAU	AAC	AAC
Asp	GAC<br>GAU			3<br>0+3	15.46<br>15.46	33.74<br>19.27	27.4<br>16.9		GAC	GAC
Cys	UGU<br>UGC		4<br>7	0+3<br>3	7.07<br>7.07	2.76<br>3.81	1.2<br>3.7		UGC	UGC
Gln	CAA<br>CAG		5.6<br>10	2<br>2	4.38<br>6.27	7.01<br>27.28	7.6<br>24.9	CAG> CAA	CAG	CAG
Glu	GAG<br>GAA	6.4<br>22		0+4<br>4	29.35<br>29.35	16.97<br>57.86	16.2<br>48.2	GAA> GAG	GAA	GAA
His	CAU<br>CAC		4<br>8	0+1<br>1	4.38<br>4.38	6.78<br>14.21	5.8<br>15.1		CAC	CAC
Lys	AAG<br>AAA			0+6<br>6	10.43<br>10.43	17.22<br>55.01	27.9<br>67.0	AAA> AAG	AAA	AAA
Phe	UUU<br>UUC		8.5<br>12	0+2<br>2	5.11<br>5.11	7.92<br>23.25	7.2<br>22.1	UUC> UUU	UUC	UUC
Tyr	UAU<br>UAC		4.3<br>8.4	0+3<br>3	4.19<br>5.04	6.72<br>16.52	4.6<br>13.3	UAC> UAU	UAC	UAC

Ile	AUA<br>AUU<br>AUC			0+2<br>0+3<br>3	[f]	0.52<br>15.79<br>43.86	0.3<br>14.1<br>40.4	AUC> AUU> AUA*	AUC	AUC
Ala	GCG<br>GCA<br>GCU<br>GCC			0+3<br>3<br>0+3<br>2	20.97<br>20.97<br>20.97<br>3.57	24.11<br>24.87<br>39.49<br>11.81	17.7<br>29.1<br>50.4<br>9.4	GCR, GCU> GCC*	GCG	GCG, GCA, GCU
Gly	GGG<br>GGA<br>GGU<br>GGC			1+1<br>1<br>0+4<br>4	11.08<br><11.08[g]<br>24.96<br>24.96	2.36<br>1.26<br>45.55<br>34.17	1.0<br>0.7<br>47.7<br>30.4	GGY> GGG> GGA*	GGC	GGU, GGC
Pro	CCG<br>CCA<br>CCU<br>CCC	5.8	2.5<br>1.6<br>8.4<br>9.6	1+1<br>1<br>0+2<br>1	5.23<br>2.56<br>6.31<br>3.75	28.82<br>5.18<br>4.38<br>1.09	22.1<br>4.4<br>4.8<br>0.4	CCR> CCY*	CCG	CCG
Thr	ACG<br>ACA<br>ACU<br>ACC			1+2<br>1<br>0+2<br>2	10.01<br>6.89<br>13.10<br>6.21	4.17<br>2.61<br>20.64<br>26.70	2.5<br>1.9<br>24.3<br>22.0	ACY> ACR*	ACC	ACU, ACC
Val	GUG<br>GUA<br>GUU<br>GUC			0+5<br>5<br>0+7<br>2	20.39<br>20.39<br>27.60<br>7.21	14.98<br>22.31<br>43.31<br>7.67	11.8<br>24.9<br>48.0<br>8.7	GUR, GUU> GUC*	GUG	GUU, GUG, GUA
Arg	AGG		-	0+1(0+2)	2.20 (5.72)	0.03	0	CGY> CGR,	CGC	CGU, CGC

	AGA<br>CGG<br>CGA<br>CGU<br>CGC	4.2	-<br>0.8<br>11.5<br>14<br>9	1<br>1<br>0+4<br>4<br>0+4	3.52<br>2.30<br>25.57<br>25.57<br>25.57	0.63<br>0.62<br>0.67<br>43.82<br>20.59	0.7<br>0.4<br>0.3<br>58.4<br>27.1	AGR*		
Leu	UUG<br>UUA<br>CUG<br>CUA<br>CUU<br>CUC		8.7<br>4.3<br>14.4<br>0.6<br>8.4<br>11	1+1<br>1<br>4+1<br>1 (1+1)<br>0+1<br>1	13.08<br>3.78<br>25.37<br>3.17<br>5.93 (9.10)<br>5.93	4.27<br>2.73<br>60.75<br>0.82<br>3.86<br>4.09	2.9<br>2.1<br>58.0<br>0.1<br>3.5<br>2.8	CUG> CUY, CUA, UUR*	CUG	CUG
Ser	AGU<br>AGC<br>UCG<br>UCA<br>UCU<br>UCC		-<br>-<br>9<br>7<br>11.6<br>14.7	1<br>0+1<br>1+1<br>1<br>0+3<br>2	5.67<br>5.67<br>8.81<br>7.36<br>11.39<br>4.03	2.19<br>9.13<br>2.51<br>1.98<br>16.33<br>11.68	2.1<br>11.6<br>0.7<br>1.1<br>17.3<br>12.0		AGC	UCU, UCC, AGC

Notes:

(a) Measured indirectly, relative to the rate of a frameshift (assumed to be codon-independent).

(b) Total number of tRNA genes coding for tRNAs that read the codon, if not all tRNAs have perfect codon-anticodon matching, the number of perfect matches+the number of non-perfect matches is given. The recognition pattern is from Dong et al (1996), the values in parenthesis for Arg and Leu include additional recognition data (Dittmar *et al*, 2005; Sorensen *et al*, 2005).

(c) At a growth rate of 2.5 doublings/hour, from (Dong *et al*, 1996).

(d) Codon frequencies, weighted by protein abundance (Dong *et al*, 1996), based on abundance data from Pedersen et al (1978)

(e) Based on tRNA concentrations and structural considerations. Asterisk marks where tRNA concentrations are invoked.

(f) Not distinguished, total concentration 24.74 μM

(g) GGA codon is read by $tRNA^{Gly2}$, measured concentration is sum of $tRNA^{Gly1}$ and $tRNA^{Gly2}$

REFERENCES

Curran JF, Yarus M (1989) Rates of aminoacyl-tRNA selection at 29 sense codons in vivo. *J Mol Biol* **209:** 65-77.

Dittmar KA, Sorensen MA, Elf J, Ehrenberg M, Pan T (2005) Selective charging of tRNA isoacceptors induced by amino-acid starvation. *EMBO Rep* **6:** 151-157.

Dong H, Nilsson L, Kurland CG (1996) Co-variation of tRNA abundance and codon usage in Escherichia coli at different growth rates. *J Mol Biol* **260:** 649-663.

Hershberg R, Petrov DA (2009) General rules for optimal codon choice. *PLoS Genet* **5:** e1000556.

Ikemura T (1981) Correlation between the abundance of Escherichia coli transfer RNAs and the occurrence of the respective codons in its protein genes: a proposal for a synonymous codon choice that is optimal for the E. coli translational system. *J Mol Biol* **151:** 389-409.

Pedersen S, Bloch PL, Reeh S, Neidhardt FC (1978) Patterns of Protein Synthesis in Escherichia Coli. Catalog of Amount of 140 Individual Proteins at Different Growth Rates. *Cell* **14:** 179-190.

Sorensen MA, Elf J, Bouakaz E, Tenson T, Sanyal S, Bjork GR, Ehrenberg M (2005) Over expression of a tRNA(Leu) isoacceptor changes charging pattern of leucine tRNAs and reveals new codon reading. *J Mol Biol* **354:** 16-24.

Sorensen MA, Pedersen S (1991) Absolute in vivo translation rates of individual codons in Escherichia coli. The two glutamic acid codons GAA and GAG are translated with a threefold difference in rate. *J Mol Biol* **222:** 265-280.
