# Supplementary material for: On Ribosome Load, Codon Bias and Protein Abundance
Source: PLoS One. 2012 Nov 7;7(11):e48542. doi: 10.1371/journal.pone.0048542 (PMC3492488; doi:10.1371/journal.pone.0048542)
Supplement: Table S2 — Correlations between measured and predicted protein abundance and between different abundance data sets. (PDF) [file pone.0048542.s006.pdf]

Table S2: Correlations between measured and predicted protein abundance and between different abundance data sets

Data sets*	Predicted abundance	Ishihama et al.	Lu et al.	Link et al.	Lopez-Campistrous et al.	Pedersen et al.
Predicted abundance	1	0.66	0.54	0.48	0.56	0.61
Ishihama et al.	0.66	1	0.62	0.43	0.66	0.69
Lu et al.	0.54	0.62	1	0.38	0.45	0.43
Link et al.	0.48	0.43	0.38	1	0.59	0.12
Lopez-Campistrous et al.	0.56	0.66	0.45	0.59	1	0.43
Pedersen et al	0.61	0.69	0.43	0.12	0.43	1

* see Table 1 for information about the data sets
