# Supplementary material for: On Ribosome Load, Codon Bias and Protein Abundance
Source: PLoS One. 2012 Nov 7;7(11):e48542. doi: 10.1371/journal.pone.0048542 (PMC3492488; doi:10.1371/journal.pone.0048542)
Supplement: Table S3 — Error estimates for the average fraction of slow codons. (PDF) [file pone.0048542.s007.pdf]

Table S3: Error estimates for the average fraction of slow codons

Range of protein abundance (Ishihama *et al*, 2008)	Number of protein sequences*	Average fraction of slow codons ($\bar{r}$)	Standard error of weighted mean over proteins ($\delta r^{(1)}$)	Standard error of fraction of all codons ($\delta r^{(2)}$)
<100	16	0.350	0.120	0.005
100-400	252	0.326	0.035	0.002
400-700	148	0.281	0.043	0.002
700-1000	87	0.285	0.060	0.003
1000-4000	158	0.214	0.039	0.002
4000-7000	30	0.155	0.090	0.004
7000-10000	13	0.134	0.119	0.007
(1-4) x 10^4	49	0.137	0.064	0.004
(4-7) x 10^4	14	0.090	0.098	0.006
(7-10) x 10^4	9	0.063	0.089	0.007
(1-4) x 10^5	10	0.071	0.087	0.007
(4-7) x 10^5	6	0.146	0.153	0.025

* Abundance ranges with only 1 or 2 sequences were excluded from the analysis.
